# Absolute Measurements of mRNA Translation in Caulobacter crescentus Reveal Important Fitness Costs of Vitamin B_12_ Scavenging

**DOI:** 10.1128/mSystems.00170-19

**Published:** 2019-05-28

**Authors:** James R. Aretakis, Alisa Gega, Jared M. Schrader

**Affiliations:** a Department of Biological Sciences, Wayne State University, Detroit, Michigan, USA; University of Copenhagen

**Keywords:** absolute quantitation, *Caulobacter crescentus*, ribosome profiling, vitamin B_12_, cell cycle

## Abstract

Caulobacter crescentus is a model for the bacterial cell cycle which culminates in asymmetric cell division, yet little is known about the absolute levels of protein synthesis of the cellular parts needed to complete the cell cycle. Here we utilize ribosome profiling to provide absolute measurements of mRNA translation in C. crescentus, providing an important resource with quantitative genome-wide measurements of protein output across individual genes. Analysis of protein synthesis rates revealed ∼4.5% of cellular protein synthesis is for genes related to vitamin B_12_ import (*btuB*) and B_12_-independent methionine biosynthesis (*metE*) when grown in common growth media lacking B_12_. While its facultative B_12_ lifestyle provides a fitness advantage in the absence of B_12_, we find that it provides a fitness disadvantage of the cells in the presence of B_12_, potentially explaining why many *Caulobacter* species have lost the *metE* gene and become obligates for B_12_.

**IMPORTANCE** Caulobacter crescentus is a model system of the bacterial cell cycle culminating in asymmetric cell division, with each daughter cell inheriting a distinct set of proteins. While a genetic network of master transcription factors coordinates the cell cycle timing of transcription for nearly 20% of *Caulobacter* genes, we lack knowledge of how many of each protein “part” encoded in the genome are synthesized. Therefore, to determine the absolute production rates across the genome, we performed ribosome profiling, providing, for the first time, a quantitative resource with measurements of each protein “part” needed to generate daughter cells. This resource furthers the goal of a systems-level understanding of the genetic network controlling asymmetric cell division. To highlight the utility of this data set, we probe the protein synthesis cost of a B_12_ utilization pathway and provide new insights into *Caulobacter*’s adaptation to its natural environments.

**Author Video**: An author video summary of this article is available.

## Abstract (precis)

Caulobacter crescentus is a model system of the bacterial cell cycle culminating in asymmetric cell division, with each daughter cell inheriting a distinct set of proteins. While a genetic network of master transcription factors coordinates the cell cycle timing of transcription for nearly 20% of *Caulobacter* genes, we lack knowledge of how many of each protein “part” encoded in the genome are synthesized. Therefore, to determine the absolute production rates across the genome, we performed ribosome profiling, providing, for the first time, a quantitative resource with measurements of each protein “part” needed to generate daughter cells. This resource furthers the goal of a systems-level understanding of the genetic network controlling asymmetric cell division. To highlight the utility of this data set, we probe the protein synthesis cost of a B_12_ utilization pathway and provide new insights into *Caulobacter*’s adaptation to its natural environments.

## INTRODUCTION

In bacterial systems biology, global mRNA translation measurements are critical for understanding how cells utilize their resources to achieve their evolutionarily selected cell growth and division cycles. To complete the bacterial cell cycle, the protein parts encoded within the genome must be transcribed into mRNAs that are translated into the appropriate number of proteins for the daughter cells to be generated. Genome-wide absolute quantitation of protein level measurements has allowed the monitoring of protein resource allocation (1, 2), revealing that these cells allocate resources for optimal growth. As the ribosome content is positively correlated with the growth rate (3), cells must optimize the fraction of protein synthesis needed to make new ribosomes (enzymes that make proteins) versus the protein synthesis needed to produce the proteomes of the daughter cells to achieve short generation times (2, 4). Optimality has also been observed at the protein-complex level, as translation of a stoichiometric amount of protein subunits to the overall multiprotein complexes has been observed (2), with different posttranscriptional strategies across species utilized to achieve the optimal protein concentration (5). Therefore, to understand the mechanisms controlling the growth and division cycles of diverse bacteria, we must understand how bacteria are able to optimize their protein synthesis resources for maximal fitness.

Caulobacter crescentus is an oligotrophic alphaproteobacterium with a carefully orchestrated cell cycle yielding asymmetric cell division and a model organism for the study of the bacterial cell cycle (6, 7). In C. crescentus, cells undergo changes in gene expression of ∼20% of their entire genome during the process of the cell cycle (8, 9). Timing of 57% of the cell cycle-regulated mRNAs is controlled at the transcription level by a master regulatory circuit that is composed of 4 transcription factors (DnaA, GcrA, CtrA, and SciP) and a DNA methylase (CcrM) (6, 10), and 49% of those cell cycle-regulated mRNAs are additionally regulated at the level of mRNA translation (8). Importantly, global C. crescentus studies have focused solely on the control of the timing of gene expression in the cell cycle, and thus, little is known about the absolute levels of protein synthesis, or how the protein synthesis resources are allocated across the proteome.

Here, we utilize ribosome profiling to achieve a quantitative genome-wide absolute measure of protein synthesis in C. crescentus. This resource provides the absolute protein synthesis rate of each protein expressed from the C. crescentus genome and a global map of protein synthesis resource allocation. Absolute levels of mRNA translation of cell cycle master regulators showed higher levels of mRNA translation compared to their known DNA binding sites for all but CcrM and a relatively low level of mRNA translation of CtrA regulatory proteins relative to the CtrA master regulator itself. PopZ, a polar protein scaffold that recruits asymmetric cell fate specification proteins (11), is at a limiting concentration compared to its client proteins, suggesting that these clients compete for access to the cell pole. Surprisingly, we discovered that the *btuB* vitamin B_12_ importer and the *metE* methionine-biosynthetic gene were among the most highly translated genes in the absence of B_12_, showing that the C. crescentus B_12_-scavenging pathway requires a surprisingly large amount of the cell’s protein synthesis resources. The high cost of protein synthesis of the B_12_-scavenging pathway is reduced in the presence of B_12_ by riboswitches in the 5′ untranslated region (UTR) of these two genes. The widely utilized lab strain NA1000 is a facultative B_12_ scavenger due to the *metE* gene, which produces methionine in the absence of B_12_, yet many natural *Caulobacter* isolates are obligate B_12_ scavengers (12). We show that the facultative B_12_-scavenging lifestyle generates a fitness tradeoff, where in the absence of B_12_ there is a positive fitness advantage from MetE’s B_12_-independent methionine production, while in B_12_’s presence there is a fitness disadvantage due to the wasted cost of MetE’s protein synthesis, providing an explanation for why many isolates have lost the *metE* gene to become obligates for B_12_.

## RESULTS

### Absolute quantitation of mRNA translation rates.

**FIG 1:**
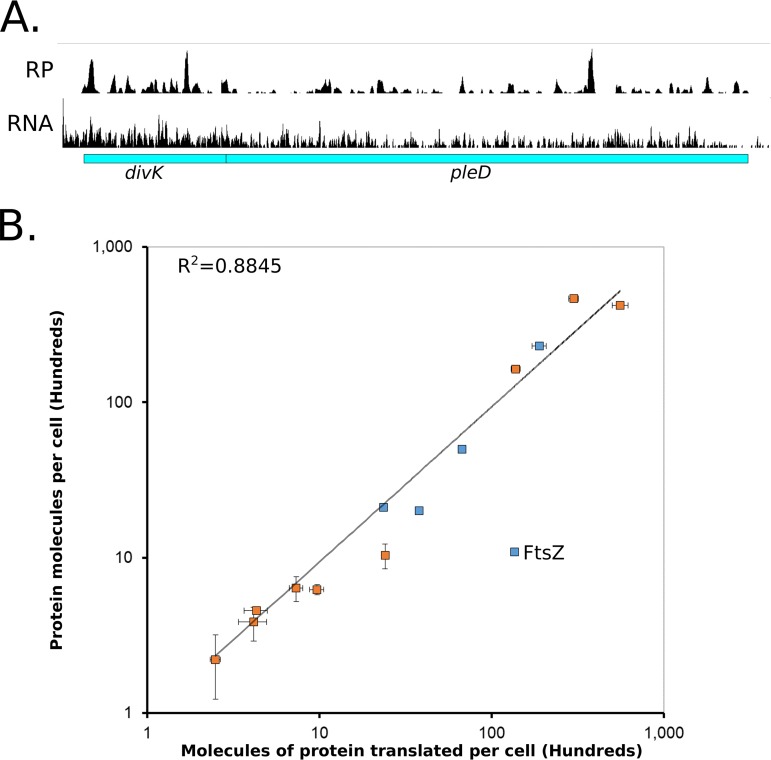
Absolute quantitation of C. crescentus protein synthesis by ribosome profiling. (A) Ribosome profiling data for cells grown in M2G medium of the *divK/pleD* operon. Average ribosome density of *divK* is 2.0 times higher than for *pleD*. mRNA data are from reference 36. (B) Absolute protein levels of unsynchronized cells measured by Western blotting (blue) or YFP fusions (orange) compared to the absolute molecules of protein translated per cell calculated by ribosome profiling. Vertical error bars indicate the standard deviation in YFP intensity or standard deviation for the Western blots, while horizontal error bars indicate the standard deviation from ribosome profiling replicates (*n* = 3). FtsZ is expected to deviate from the line since its protein levels are under proteolytic control (17). Data are in Tables S1 and S2.

Ribosome profiling provides a global direct measure of the protein synthesis rate by sequencing ribosome-protected mRNA footprints (2, 13, 14). To determine absolute rates of translation in C. crescentus, ribosome profiling was performed in unsynchronized C. crescentus NA1000 cells grown in M2G minimal medium. In fast-growing bacteria where the rate of translation is the main driving force of protein levels, protein degradation can be negligible, and therefore, the main driving force of protein levels is mRNA translation (2). This is largely true in C. crescentus, as >95% of proteins were found to have half-lives longer than the cell cycle (15). First, we examined the ribosome footprint density along each open reading frame (ORF) on mRNAs as a relative measure for translation (Fig. 1A). For example, in the *divK/pleD* polycistronic operon, we find that *divK* has 2.0-fold-higher ribosome density than *pleD* (Fig. 1A). For absolute quantitation, it is assumed that the average elongation rate is constant for each mRNA, which would allow the average ribosome density to be directly proportional to the rate of protein synthesis of each ORF (2). It is also assumed that all ribosomes will finish translation and make the full-length protein (2). Next, to reduce the impact of fast- and slow-moving ribosomes in the ribosome occupancy profiles along ORFs on the quantitative level of translation, we used winsorization to correct the average ribosome footprint density of each ORF (see Table S1 in the supplemental material). Start codon and stop codon regions were omitted from the analysis to avoid biases in slow-moving ribosomes that are initiating or terminating (13, 16).

10.1128/mSystems.00170-19.4 TABLE S1 Absolute measurements of mRNA translation for M2G and PYE. Download Table S1, XLSX file, 0.7 MB. Copyright © 2019 Aretakis et al. 2019 Aretakis et al. This content is distributed under the terms of the Creative Commons Attribution 4.0 International license.

10.1128/mSystems.00170-19.5 TABLE S2 Average molecules of protein translated per cell and average proteins per cell. *^a^*Ribosome profiling data from reference 36. Download Table S2, DOCX file, 0.01 MB. Copyright © 2019 Aretakis et al. 2019 Aretakis et al. This content is distributed under the terms of the Creative Commons Attribution 4.0 International license.

To convert ribosome density to absolute mRNA translation rates, we measured the average protein mass of C. crescentus cells, which was multiplied by the fractional ribosome density measurement of each gene and divided by the molecular weight (*k_i_* = ϕ*_i_P*/*mW_i_*) (2). This measure of the average number of proteins translated per cell correlated well between the protein concentrations reported in the literature as well as protein concentration measurements reported here using yellow fluorescent protein (YFP) intensity of C-terminally tagged gene fusions (Fig. 1B; Fig. S1 and Table S2). As expected, FtsZ, the key cell division protein which is known to be a substrate of cell cycle-dependent proteolysis (17), has a 13-fold-larger amount of translated protein than protein observed in the cell, while stable proteins ranged between 0.65- and 2.3-fold. The same phenomenon of higher translation levels than protein levels was also observed for the proteolyzed cell cycle regulators DnaA and CcrM, and upon deletion of the Lon protease, which is known to be responsible for their proteolysis, the correlation was restored (Peter Chien, personal communication) (Fig. S1) (18, 19). These data suggest that the absolute measures of mRNA translation are reflecting the absolute protein synthesis rate for each ORF in C. crescentus and provide a reasonable measure of steady-state protein levels for stable proteins (data can be found in Table S1).

10.1128/mSystems.00170-19.1 FIG S1 Absolute protein levels of unsynchronized cells in PYE medium measured by Western blotting (blue) or YFP fusions (orange) compared to the absolute molecules of protein translated per cell calculated by ribosome profiling. PYE ribosome profiling data were collected in reference 36. Vertical error bars indicate the standard deviation in YFP intensity or standard deviation for the Western blots. FtsZ, CcrM, and DnaA are indicated as their protein levels are under proteolytic control by Lon protease (18, 19). CcrM and DnaA protein levels in a strain lacking their protease, Lon, are indicated in dark blue (Peter Chien, personal communication). Data are in Tables S1 and S2. Download FIG S1, TIF file, 0.8 MB. Copyright © 2019 Aretakis et al. 2019 Aretakis et al. This content is distributed under the terms of the Creative Commons Attribution 4.0 International license.

### Global analysis of C. crescentus absolute mRNA translation levels.

**FIG 2:**
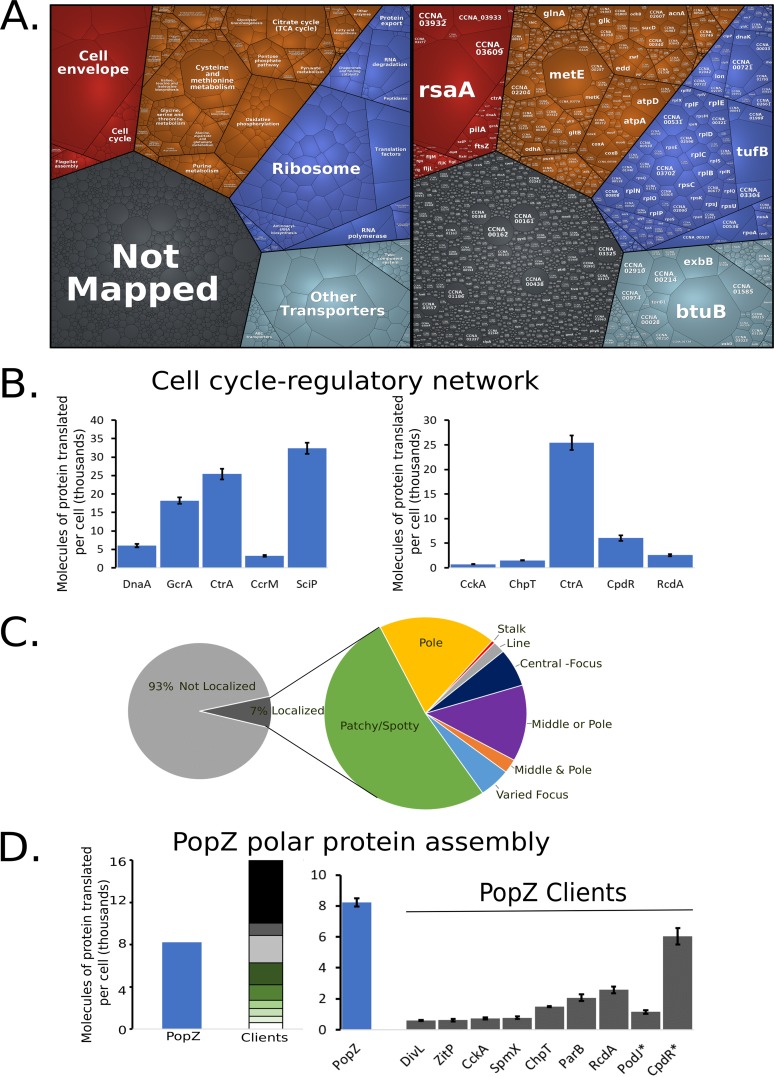
Global analysis of C. crescentus protein synthesis. (A) Proteomap with each polygon representing a single gene with area scaled to the fraction of ribosome-protected mRNA footprints measured. Red is cellular processes, orange is metabolism, blue is genetic information processing, light blue is environmental information processing, and gray is genes of unknown function. (B) Molecules of protein translated per cell for the cell cycle master regulators (left) and CtrA regulatory network (right). (C) (Left) Fraction of ribosome-protected mRNA footprints encoding localized (dark gray) or nonlocalized (light gray) proteins. (Right) Zoomed-in analysis of the fraction of proteins with different subcellular localization patterns based on reference 26. (D) Polar protein competition. (Left) Molecules of protein translated per cell for the polar protein scaffold PopZ and its known clients (27–29). Proteins with known proteolysis are highlighted with asterisks.

C. crescentus cells dedicate a significant percentage of their protein synthesis to several major cellular processes associated with cell growth (Fig. 2A; Fig. S2). Across major KEGG categories, we analyzed the percentage of ribosome footprints to understand their allocation of protein synthesis capacity (Table S3). Nutrient transporters (11.8%), the ribosome (11.2%), and the cell envelope (9.8%) represent the largest classes of protein production for C. crescentus. A significant fraction of protein synthesis capacity (25.5%) is allocated to produce proteins of unknown function, showing that a significant fraction of the cell’s protein synthesis capacity is not understood. By comparing the fraction of the translation KEGG category across minimal medium (M2G) (15.9%) and a richer complex medium (peptone-yeast extract [PYE]) (22.8%), we find that the cells dedicate a larger amount of protein synthesis capacity to making translational machinery in rich medium, similar to Escherichia coli (3). Additionally, we see that a larger amount of protein synthesis capacity in “cell growth and death” is observed in M2G (11.3%) than in PYE (6.45%), owing largely to increased protein synthesis capacity of an operon of cell-contact-dependent toxins and immunity proteins that are known to be expressed in stationary phase (Fig. S2) (20).

10.1128/mSystems.00170-19.2 FIG S2 PYE fractional protein synthesis Proteomap. Global analysis of C. crescentus protein synthesis in PYE and M2G media. (A) (Left) Proteomap of cells grown in PYE medium with the area scaled to the fraction of ribosome-protected mRNA footprints measured. (Right) Proteomap of cells grown in M2G medium shown at the same level as cells grown in PYE medium with KEGG categories (67). (B) Fraction of ribosome-protected mRNA footprints compared to gene essentiality as determined in reference 69. Red is essential genes, blue is nonessential, yellow is high-fitness genes, and gray is genes that were not determined (69). (C) (Left) Fraction of ribosome-protected mRNA footprints in localized (dark gray) or nonlocalized (light gray) mRNAs for cells grown in PYE medium. (Right) Zoomed-in analysis of the fraction of proteins with different subcellular localization patterns as determined in reference 26. Download FIG S2, TIF file, 2.8 MB. Copyright © 2019 Aretakis et al. 2019 Aretakis et al. This content is distributed under the terms of the Creative Commons Attribution 4.0 International license.

10.1128/mSystems.00170-19.6 TABLE S3 Fraction of total protein synthesis for each KEGG category. *^a^*KEGG category data from reference 70. Download Table S3, DOCX file, 0.01 MB. Copyright © 2019 Aretakis et al. 2019 Aretakis et al. This content is distributed under the terms of the Creative Commons Attribution 4.0 International license.

Interestingly, while the cell cycle is a major area of study in C. crescentus, the cell cycle genes represent only a small fraction of protein synthesis capacity (1.78%), and yet these genes play a critical role in shaping the growth and division cycles. The cell cycle-regulatory circuit itself is composed of four transcription factors (DnaA, GcrA, CtrA, and SciP) and a DNA methylase (CcrM) whose spatiotemporal activation facilitates cell cycle progression (6). For master regulator proteins, the cell produces between ∼3,000 and 30,000 copies of each protein, which corresponds to between 71- and 648-fold more proteins than the number of known DNA binding sites that they control (10), with the exception of CcrM (Table S4). Three thousand two hundred eighty CcrM proteins are translated to methylate the 4,542 GANTC sites per chromosome (Fig. 2B). While the number of CcrM proteins is approximately one-third the number of GANTC sites present after DNA replication, CcrM is a processive enzyme (21), suggesting that each CcrM may on average methylate ∼3 GANTC sites. While the number of CtrA proteins translated (25,400 proteins) corresponds closely with the amount measured in predivisional cells (18,000 to 22,000 [22, 23]), CtrA is produced at a significantly higher level than its collection of regulatory kinases, phosphotransferases, and proteolytic adapters that control its cell cycle-dependent activity (Fig. 2B). GcrA interacts with the RNA polymerase/σ^70^ complex to activate transcription of target promoters (24), where an ∼4-fold excess of GcrA over σ^70^ is produced, suggesting that excess GcrA may accelerate binding to the RNA polymerase holoenzyme (25) to facilitate subsequent recruitment of σ^70^.

10.1128/mSystems.00170-19.7 TABLE S4 Comparison of absolute translation level to DNA binding sites. *^a^*Number of binding sites were summed together from reference 10 based on data from references 24 and [Bibr B71][Bibr B72][Bibr B75]. Download Table S4, DOCX file, 0.02 MB. Copyright © 2019 Aretakis et al. 2019 Aretakis et al. This content is distributed under the terms of the Creative Commons Attribution 4.0 International license.

As many proteins were found to have distinct subcellular patterns of protein accumulation in C. crescentus as determined in reference 26, we compared protein synthesis capacity to the localization patterns of proteins observed in this data set (Fig. 2C). Seven percent of protein synthesis occurs for “localized proteins” in C. crescentus. Of those localized proteins, most are “patchy/spotty,” while a significant fraction has a subcellular address where the protein accumulates (pole, stalk, or center) (Fig. 2C). Many proteins are specifically required to form asymmetric polar protein complexes that function to determine cell fate upon division (6). Many of these polarly localized proteins are recruited to the cell pole through the multimeric hub protein PopZ (27–29). Interestingly, by examining PopZ and its known client proteins, we find that PopZ is made in limiting amounts (Fig. 2D), suggesting that the clients compete for PopZ binding *in vivo*.

### Analysis of vitamin B_12_ and methionine metabolism.

**FIG 3:**
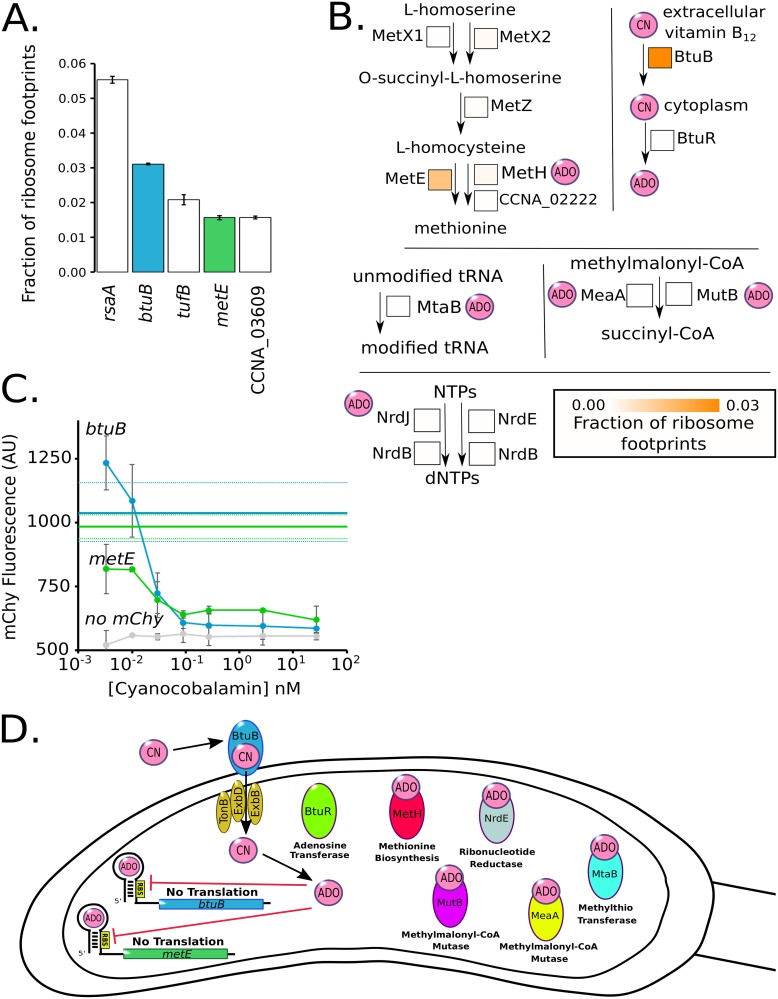
C. crescentus cells are starved for B_12_ in laboratory growth medium. (A) Fraction of ribosome footprints for the most highly translated mRNAs in M2G. B_12_-related genes are colored. (B) Pathway of methionine biosynthesis, MetX1 (CCNA_03309), MetX2 (CCNA_00559), MetZ (CCNA_02321), MetE (CCNA_00515), MetH (CCNA_02221), and CCNA_02222. Pathway for B_12_ utilization, BtuB (CCNA_01826) and BtuR (CCNA_02321). Pathway for tRNA modification, MtaB (CCNA_03798). Pathway for nucleotide reduction, NrdJ (CCNA_01966), NrdE (CCNA_03607), and NrdB (CCNA_00261). Pathway for succinyl-CoA biosynthesis, MeaA (CCNA_03177) and MutB (CCNA_02459). Square boxes next to each enzyme contain an orange heat map which represents the fraction of ribosome footprints. ADO (adenosyl) and CN (cyano) refer to the B_12_ upper ligand. (C) Negative regulation by B_12_ riboswitches on the *btuB* and *metE* genes. Translation reporters for the *btuB* and *metE* genes fused to mCherry assayed in M2G with the indicated concentrations of B_12_. Error bars represent standard deviation for mCherry fluorescence in three biological replicates of the B_12_ dilution series (*n* = 3). Solid blue and green horizontal lines indicate the mChy fluorescence without vitamin B_12_ for *btuB* and *metE,* respectively, and dashed lines indicate the standard deviation. (D) Cartoon of B_12_-regulated pathways in C. crescentus. *btuB* and *metE* genes contain negative regulatory B_12_ riboswitches. BtuB and BtuR are part of the B_12_ import and utilization pathway. MetH, NrdE, MeaA, MutB, and MtaB are B_12_-dependent enzymes for methionine biosynthesis.

Analysis of the most highly translated proteins found that RsaA, the surface layer protein, was the most highly translated protein in the cell (Fig. 3A) (30). Elongation factor Tu was the third most abundant cytoplasmic protein owing to its requirement to deliver aminoacyl-tRNAs to the ribosome during translation (31). Surprisingly, we also observed that the homolog of the B_12_ importer (*btuB*, second highest) and the methionine-biosynthetic gene (*metE*, fourth highest) were among the most highly translated proteins. Vitamin B_12_ is an important enzymatic cofactor that in C. crescentus is used for the biosynthesis of methionine, deoxynucleoside triphosphate (dNTP) production, tRNA modification, and isomerization of methylmalonyl coenzyme A (CoA) to succinyl-CoA (Fig. 3) (32). C. crescentus cannot synthesize B_12_ *de novo* but can import it through the BtuB protein (33). In the cytoplasm, both MetE and MetH perform the rate-limiting step of methionine biosynthesis, where MetH requires B_12_ but has a higher specific activity than MetE (Fig. 3B) (34, 35). Of note, both BtuB and MetE are translated at much higher levels than the other components related to methionine biosynthesis (Fig. 3B). Both the *btuB* and *metE* genes are the only two genes in C. crescentus with B_12_ riboswitches encoded in their 5′ UTRs (36). We tested the function of these riboswitches by creating 5′ UTR fusions to the mCherry gene driven by the vanillate promoter and subjecting the cells to various concentrations of B_12_ in the form of cyanocobalamin (Fig. 3C). Both the *metE* and *btuB* 5′ UTR reporters showed high translation in the absence of B_12_ and exhibited a B_12_ concentration-dependent translational shutoff (Fig. 3C). The *metE* riboswitch appears to be more sensitive to B_12_ concentration, with a *K*_1/2_ of 0.062 nM, while the *btuB* riboswitch *K*_1/2_ was 0.19 nM, both in line with the concentrations found in aquatic ecosystems (Fig. S3) (37, 38). Taken together, these data show that C. crescentus cells are investing a large amount of their protein synthesis capacity toward B_12_ uptake and the B_12_-independent methionine pathway in the absence of B_12_. We therefore hypothesized that the cells are wasting energy in the absence of B_12_ by producing these very costly proteins.

10.1128/mSystems.00170-19.3 FIG S3 Nonlinear curve fit for B_12_-dependent riboswitch repression. mCherry intensities at each cyanocobalamin concentration were fitted to a Michaelis-Menten equation using QTIplot software to determine the *K*_1/2_ of cyanocobalamin. *R*^2^ values were 0.95 for *metE* (green) and 0.85 for *btuB* (blue). Download FIG S3, TIF file, 0.7 MB. Copyright © 2019 Aretakis et al. 2019 Aretakis et al. This content is distributed under the terms of the Creative Commons Attribution 4.0 International license.

**FIG 4:**
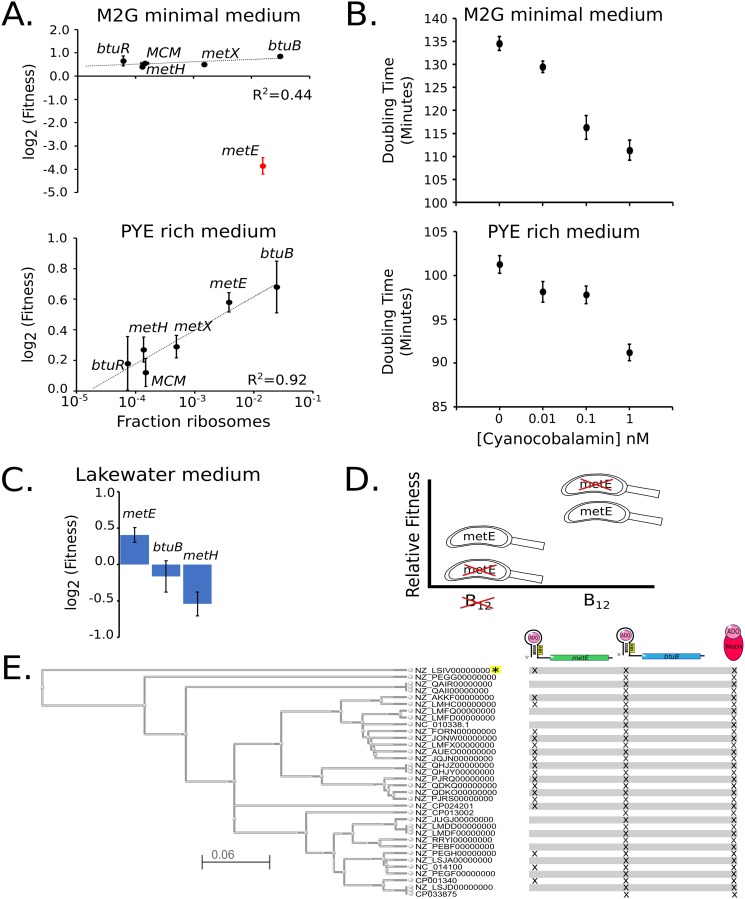
Excess protein synthesis rates for methionine-biosynthetic genes correlate with fitness cost. (A) Protein synthesis cost measured in the fraction of ribosomes (Table S1) on the *x* axis versus the Tn-seq-derived fitness values for the *btuB* B_12_ importer and methionine-biosynthetic genes under growth in minimal or rich medium as measured previously (39) (biological replicates, *n* = 2 for M2G, *n* = 10 for PYE). Black points represent nonessential genes for methionine biosynthesis, and red points represent genes required for methionine biosynthesis under the specified growth condition. Error bars represent standard deviation. Curve fits were performed only on nonessential genes. (B) Doubling times of C. crescentus cells in M2G and PYE media with indicated concentrations of B_12_. Error bars represent the standard deviation for doubling time measurements (biological replicates, *n* = 3 for each condition). (C) Tn-seq-derived fitness values for *metE*, *btuB*, and *metH* under growth in Lake Michigan lake water as measured previously (57). (D) Fitness tradeoff of facultative versus obligate B_12_ scavenging. Relative fitness shown for species with *metE* (facultative) or without *metE* (obligate) in environments lacking or containing sufficient B_12_. (E) Phylogenetic tree of all *Caulobacter* species with completed genomes based on *btuB* and *metH* protein sequences. Each species is labeled by its NCBI accession identifier, and the scale represents the Kimura distance. Marks next to species represent the presence of a *metE*, *btuB*, or *metH* gene. All species have a predicted B_12_ riboswitch upstream of *btuB* and *metE* genes (not shown), except the species noted with the yellow asterisk. A list of species names can be found in Table S7.

To assess if the cells are wasting energy from the B_12_-related pathways, we examined the fitness of C. crescentus cells with disruptions in the nonessential components of these pathways in the absence of B_12_ (Fig. 4A). For this, we used published transposon sequencing (Tn-seq) data sets (39) and analyzed B_12_-related genes whose disruption would not have polarity effects (single genes or last genes in operons) in M2G minimal medium or PYE rich medium, neither of which contains B_12_ (12, 40). In M2G minimal medium, cells require the *metE* gene to make methionine (33), while the other nonessential B_12_-related components showed increases in fitness when disrupted that were proportional to their protein synthesis costs (Fig. 4A). In PYE rich medium, which contains methionine in the peptone, the *metE* gene is no longer essential, but instead, its disruption leads to higher fitness (39). Indeed, all the components of the methionine pathway led to increases in fitness proportional to their protein synthesis cost when disrupted in PYE (Fig. 4A) (39). These data show that in the absence of B_12_, the excessive translation of these proteins leads to unnecessary costs of protein synthesis that limit the fitness of C. crescentus cells.

To test the effects of B_12_ on C. crescentus cell growth, we examined the growth rate of cells cultured in M2G medium containing B_12_ in the form of cyanocobalamin. Here, we observe faster growth in a B_12_ concentration-dependent manner (Fig. 4B; Table S5), with up to a 21% faster doubling time observed at 1 nM B_12_ in M2G medium. Eleven percent acceleration of cell growth rates also occurs in PYE medium, which contains methionine in the peptone, suggesting that the growth enhancement of B_12_ is likely caused in part by reduced protein synthesis costs from *btuB* and *metE* riboswitches. We attempted to separate B_12_’s effects on methionine synthesis from its effects on other pathways by addition of exogenous methionine in the presence or absence of B_12_, but exogenous methionine leads to a dramatic decrease in growth by an unknown mechanism (41). Overall, we find that B_12_ significantly enhances the growth rate of NA1000 cells.

10.1128/mSystems.00170-19.8 TABLE S5 Doubling time measurements at different concentrations of cyanocobalamin. Download Table S5, DOCX file, 0.01 MB. Copyright © 2019 Aretakis et al. 2019 Aretakis et al. This content is distributed under the terms of the Creative Commons Attribution 4.0 International license.

## DISCUSSION

### Absolute quantitation of protein synthesis in C. crescentus.

As the cost of protein synthesis is a significant output of the cell’s energy, absolute quantitation of translation is a powerful method to measure gene expression and resource allocation. Here, we present an absolute protein synthesis resource for C. crescentus generated by ribosome profiling which will be vital for systems modeling efforts for the C. crescentus cell cycle (42–45) and in the subsequent optimization of synthetic *Caulobacter* genomes (46, 47). Across the proteome, the mRNA translation resource allocation showed that 1.4% of the translation machinery is dedicated to translation of cell cycle-regulatory genes. We observed that for all the cell cycle master regulators, except CcrM, the number of proteins translated dramatically exceeds the total number of DNA binding sites in the genome (Fig. 2B). We hypothesize that the high concentrations of these factors may facilitate rapid activation of target gene transcription during each phase of the cell cycle.

Approximately 7% of protein synthesis capacity is dedicated to genes whose protein products were found to be localized in one of several modes of subcellular organization (Fig. 2C) (26). Recent reports suggest that some of these foci are formed by liquid-liquid phase separation of the proteins into membraneless organelles (48, 49). For C. crescentus BR bodies, the concentration of the condensate-forming protein RNase E (6.3 μM) appears to correspond closely to the transition boundary for liquid-liquid phase separation (48), potentially allowing control of the assembly of these bodies. Interestingly, we observed that the polar protein scaffold PopZ, which facilitates recruitment of asymmetrically localized signaling proteins to the cell poles (11, 27, 29), is present at approximately one-half the concentration of its client proteins, suggesting that clients compete for PopZ access (Fig. 2D). Dynamic competition of clients for PopZ may be important for the ordered assembly of unique proteins at each cell pole and may impact the spatial activation of downstream signaling outputs (27, 50, 51).

### Implications of B_12_-scavenging pathway for environmental fitness.

B_12_ is an important enzymatic cofactor that is required for the activity of enzymes involved in biosynthesis of methionine, dNTP production, tRNA modification, and isomerization of methylmalonyl-CoA to succinyl-CoA (Fig. 3B and D) (32). Like many bacteria, C. crescentus cannot produce B_12_ but can scavenge it from the environment (33), where it can increase the growth rate of C. crescentus by up to 21% (Fig. 4B). *btuB*, the B_12_ importer, and *metE*, the B_12_-independent methionine synthase, are among the most highly expressed genes, accounting for ∼4.5% of all protein synthesis capacity (Fig. 3A). To counteract the protein synthesis demand, *btuB* and *metE* genes also contain B_12_ riboswitches that reduce translation when sufficient B_12_ enters the cytoplasm (Fig. 3C). Freshwater bodies typically have B_12_ concentrations in the range from 0.11 nM to below the level of detection (<0.1 pM) (37, 38, 52–55), suggesting that high levels of BtuB may help facilitate import. Importantly, the conserved *metE* and *btuB* riboswitches are sensitive to B_12_ in physiologically relevant ranges (*K*_1/2_ = 0.062 nM and 0.19 nM, respectively), and their different sensitivities suggest that *metE* translation would be downregulated before shutting off the *btuB* importer. At high concentrations of B_12_ that saturate the riboswitches (Fig. 4B), a significant portion of the increase in growth rate is likely due to the liberation of protein synthesis resources on these two highly expressed genes (Fig. 3A and C). When grown in M2G or PYE medium lacking B_12_, disrupting the *btuB* gene increases fitness by freeing up wasted protein synthesis resources (Fig. 4A) (39). Similarly, *metE* disruption leads to increased fitness in PYE, which contains methionine, but *metE* disruption becomes essential in M2G minimal medium as it is required to make methionine (Fig. 4A) (39). Why then does C. crescentus have a facultative B_12_ lifestyle containing both B_12_-dependent and -independent methionine biosynthesis pathways?

Perhaps B_12_-independent and B_12_-dependent pathways exist to buffer fluctuations in environmental B_12_ concentrations. The concentration of available B_12_ in a freshwater body shows variation of up to 40-fold between different sampling locations and at the same sampling location at different times (38, 52–56). Having both methionine biosynthesis pathways adds flexibility to generate methionine under either high- or low-B_12_ conditions; however, these pathways have different protein synthesis costs. The B_12_-independent pathway requires 1.57% of the total protein synthesis capacity to make sufficient MetE, while the B_12_-dependent MetH pathway uses only 0.156% (Fig. 3B). Interestingly, disrupting the *metE* gene in lake water leads to a fitness advantage; however, disrupting *btuB* or *metH* leads to a fitness decrease as determined in reference 57 (Fig. 4C). Although not measured directly, the gene fitness signature from the experiment leads us to infer that physiologically relevant B_12_ concentrations were present in the sampled lake water (57). The increased fitness of *metE* disruptions in lake water suggests that increased biosynthetic flexibility comes with a negative fitness cost from protein synthesis resources wasted on MetE (Fig. 4D). Forty-seven percent of fully sequenced *Caulobacter* species have lost the *metE* gene but not the *btuB* and *metH* genes, suggesting that the observed environmental fluctuations in B_12_ concentration (38, 52) alter the selective pressure on *metE* (Fig. 4E).

Surprisingly, a recent survey of available metagenomic 16S rRNA sequencing data showed that *Caulobacter* is more abundant in soil/compost than in aquatic ecosystems (58). Soil has been shown to have B_12_ levels that can range from 20 nM to 0.3 nM, correlated with levels of organic matter (59), while bodies of freshwater typically have B_12_ concentrations in the range of 0.11 nM to below the level of detection (0.1 pM) (37, 38, 53–56). The higher B_12_ concentration in soil will enhance the growth rate and may explain the increase in relative abundance in this environment.

## MATERIALS AND METHODS

### Bacterial strains and cell growth.

A list of all bacterial strains used here can be found in Table S6 in the supplemental material (76–81). C. crescentus cells were grown in M2G or PYE growth medium (40) and supplemented with the appropriate antibiotic concentrations (60). E. coli cells used for cloning were grown in LB medium and supplemented with the appropriate antibiotics.

10.1128/mSystems.00170-19.9 TABLE S6 List of bacterial strains. Download Table S6, DOCX file, 0.02 MB. Copyright © 2019 Aretakis et al. 2019 Aretakis et al. This content is distributed under the terms of the Creative Commons Attribution 4.0 International license.

10.1128/mSystems.00170-19.10 TABLE S7 Table of accession numbers and organism names from Fig. 4. *^a^*Accession numbers and names were taken from the NCBI GenBank database. Download Table S7, DOCX file, 0.01 MB. Copyright © 2019 Aretakis et al. 2019 Aretakis et al. This content is distributed under the terms of the Creative Commons Attribution 4.0 International license.

### Ribosome profiling.

Ribosome profiling was performed similarly to procedures in references 8 and 36, except that contaminating rRNA fragments generated during micrococcal nuclease (MNase) digestion were depleted to allow deeper quantitation of resulting mRNA translation similarly to reference 2. For a detailed protocol for the procedure, see reference 16. Five hundred milliliters of NA1000 cells were grown in M2G medium to an OD_600_ of 0.5, treated with 100 μg/ml chloramphenicol for 2 min, and then harvested by centrifugation and flash-frozen in liquid nitrogen. Cells were then lysed on a mixer mill (Retsch mm400) for 6 cycles of 3 min at 15 Hz and thawed, membranes were pelleted, and the supernatant was footprinted by addition of MNase (Roche). After footprinting, MNase was quenched with EGTA, and samples were separated by sucrose gradient fractionation. 70S peaks were purified, phenol chloroform extracted, and ethanol precipitated (16). Resulting mRNA fragments were size selected by 10% acrylamide-1× TBE-7 M urea PAGE, end repaired, 3′ adapter ligated, reverse transcribed, circularized, and depleted of rRNA fragments (2, 14, 16). rRNA cDNA fragments were removed using biotin-linked DNA oligonucleotides (oCaulo1, 5′/5Biosg/CGCTTACGGGGCTATCACCCA; oCaulo2, 5′/5Biosg/TGGCAACTAATCACGAGGGTT; oCaulo3, 5′/5Biosg/CTCATCTGGTTGCCCAAAAGA; oCaulo4, 5′/5Biosg/TGGTTCAGGAATATTCACCTG) and MyOne streptavidin C1 Dynabeads (Invitrogen) as in reference 2. Resulting circular cDNAs were amplified by PCR using Phusion DNA polymerase (Fermentas) with indexing primers (61), pooled, and sequenced on an Illumina HiSeq 2000. Data for three ribosome profiling replicates were deposited in the Gene Expression Omnibus under accession number GSE126485. The three M2G replicates were further analyzed together with a PYE data set collected previously (36).

Ribosome footprint reads were mapped to the genome as center-weighted reads (13), and extremely fast and slow codons were corrected for by winsorization of the bottom 5% and top 95% of nucleotides, respectively. The resulting fraction of ribosome footprints (ϕ*_i_*) of each gene (*i*) compared to the total ribosome footprint total was calculated and converted into the number of molecules of protein translated per cell (*k_i_*) by the equation *k_i_* = ϕ*_i_P*/*mW_i_*, where *P* is the average protein mass per cell and *mW_i_* is the protein product’s molecular weight as originally described in reference 2. Average protein mass per cell (*P*) was measured as follows. Five-milliliter mid-log cultures of NA1000 cells were grown in M2G or PYE medium overnight in a roller wheel at 28°C. Once the cells reached an OD_600_ of 0.3, a 100-μl aliquot of the cells was diluted and counted on PYE plates to measure the number of viable cells and another aliquot was saved for protein concentration measurements. To measure protein content, 100 μl cells was spun down in a microcentrifuge at 14,000 rpm for 30 s, the supernatant was removed, and the remaining cell pellets were resuspended in 100 μl of 1× Laemmli sample buffer lacking any dyes. After resuspension, samples were boiled for 5 min at 95°C and then placed on ice. Lysate protein concentrations were measured using the Pierce 660-nm protein assay (Thermo Fisher) and with comparison of the lysate *A*_600_ with a linear curve fit of bovine serum albumin (BSA) standards. The mass of protein from the sample was then divided by the number of viable cells to yield the average protein mass/cell. The resulting average protein masses per cell were (492 ± 170) × 10^−15^ g/cell in M2G and (519 ± 228) × 10^−15^ g/cell in PYE.

### Absolute quantitation of C-terminal YFP fusions.

Five-milliliter mid-log cultures of NA1000 cells harboring C-terminal eYFP fusions (gift of the Shapiro Lab, Stanford University) were grown in M2G or PYE medium overnight in a roller wheel at 28°C. Once the cells reached an OD_600_ of 0.3, cells were spotted on M2G agarose pads for imaging. Images were collected on a Leica DM6000B microscope with a Hamamatsu C9100 electron multiplying charge-coupled device (EMCCD) camera and a 100× PH3 Plan Apo 1.40-numerical-aperture (NA) objective with in a Semrock model 2427A YFP filter cube with 100-ms exposure time. Fluorescence intensity was quantified using ImageJ by segmenting the cells and measuring the average pixel intensity of the cell area. Background intensity was subtracted using the NA1000 average YFP pixel intensity. For each fusion strain, a minimum of 50 cells were used for the analysis with a minimum of two technical replicates. As MipZ molecules per cell had been previously measured (62) by quantitative Western blotting, we converted the MipZ-YFP pixel intensity to the number of molecules/cell and multiplied this conversion factor by the YFP intensities of all other C-terminal YFP fusions. The average across replicates and the standard deviation (σ) are reported in Table S2.

### Doubling time measurements.

Treatments were started from log-phase cultures grown overnight in the absence of cyanocobalamin (Sigma-Aldrich) and diluted in fresh medium to an OD_600_ of 0.05. Each treatment was split into a separate flask, and the correct amount of cyanocobalamin was added to the concentrations of 1 nM, 0.1 nM, 0.01 nM, and 0 nM. Fifty milliliters from each treatment was added to three different 250-ml Kimex flasks, for three replicates of each of the four treatments. An initial OD_600_ measurement was taken of each replicate using a cuvette and a NanoDrop spectrophotometer. The 12 250-ml flasks were then placed in a 28°C shaker incubator at 250 rpm. OD_600_ time points of each flask were taken throughout the logarithmic growth phase. An exponential regression of the log-phase time points was used to calculate the doubling time of each replicate.

### Translation reporter assay.

JS417, JS423, and JS440 strains were started from log-phase cultures grown overnight in the absence of cyanocobalamin and diluted in medium with vanillate and antibiotic to an OD_600_ of 0.05. A dilution series of each strain was used to fill tubes with 2 ml of culture at each cyanocobalamin concentration: 27 nM, 2.7 nM, 0.9 nM, 0.3 nM, 0.1 nM, 0.033 nM, and 0 nM. The 21 2-ml-cultures were then grown and induced over an 8-h period by placing the tubes in a 28°C shaker incubator at 250 rpm for 8 h. After 8 h, 2 μl from each culture was pipetted onto an M2G-agarose pad on a microscope slide. Each treatment was imaged on a microscope using both phase contrast and an mChy filter cube. Average fluorescent intensities were calculated using MicrobeJ (63) across a minimum of 100 cells.

### B_12_ homolog identification and phylogenetic tree mapping.

Protein sequences for *btuB*, *metH*, and *metE* were determined for each *Caulobacter* species with a complete genome by using the NCBI Basic Local Alignment Search Tool (BLAST) with default settings by searching the protein sequence of each NA1000 gene for homologs with an E score of <10^−19^ (64). The *btuB* and *metH* genes were then used to generate the phylogenetic tree using the NCBI Genome Workbench software and the MUSCLE multiple sequence alignment package (65). The tree is a maximum likelihood generated with the default settings from MUSCLE. Riboswitches were identified using rfam (66) and by searching the upstream regions of *btuB* and *metE* genes (up to 1,000 bp upstream of their predicted operons).

### Proteomap generation.

Categories were taken from predefined Kyoto Encyclopedia of Genes and Genomes (KEGG) categories (67). Categories were then ranked based on priority, and any gene that might have been present in more than one category was deleted from those with lower priority. The 200 most numerous proteins were then hand checked. Any that had not been automatically assigned to a KEGG category were compared against other organisms to place them in their most appropriate category. The categorized genes along with the ribosome profiling data were used to create the Proteomap (68).

### Strain construction. (i) JS417.

The insert *btuB_*5′UTR was generated by IDT as a gBlock construct for the +1 transcription start site (TSS) through the start codon of the *btuB* gene. The *btuB_*5′UTR gBlock had the sequence AAGCGTTCAATTGGATCCAATCTTGACGTCCGTTTGATTACGATCAAGATTGGATCCAGCGTCAGGTTCCTCGAAAGAGGATGAAAAGGGAACGAGGTTGAAGACCTCGGCTGCCCCCGCAACTGTAAGCGGCGAGCTTCGCGTCACATGCCACTGGGCCCAAAAGGCCTGGGAAGGCGACGCCCAGAAGCATTGACCCGTGAGCCAGGAGACCTGCCCGGCGCAGTCGTTCATCGCTCGGCCGGGGTGCGCCGAACGAACGGGATCTCCCGAGAAACGACAGTCAACAGGCCGCGCGACGGCCTGAGCGTCCGCGTCTTCGCGGGCGGTCGGGAGGTCGCGTGGGTCGTTCATAACGGGAAGACTGTATTATGTTAATTAATATGCATGGTAC.

The plasmid pRVChyC-2 (60) was cut with MfeI and PacI, and the gBlock segment *btuB_*5′UTR was inserted into the plasmid by Gibson assembly. Next, the resulting plasmid was transformed into E. coli DH5α cells and selected on LB-kanamycin (Kan) plates. The resulting Kan^r^ colonies were then screened by PCR for the insert and verified by Sanger sequencing (Genewiz). The purified plasmid was then transformed into NA1000 cells by electroporation and plated on PYE-Kan plates. The resulting colonies were screened for mChy fluorescence after induction with vanillate.

**(ii) JS423.** The insert *metE_*5′UTR was generated by IDT as a gBlock construct for the +1 TSS through the start codon of *metE*. The *metE_*5′UTR gBlock had the sequence AAGCGTTCAATTGGATCCAATCTTGACGTCCGTTTGATTACGATCAAGATTGGATCCAGTCGTGGTCTGCGGACGTTCGCGTCCGGAGCTAAGAGGGAAGTCGGTGAGGGCGTGAAACCCTGAATCCGGCGCTGCCCCCGCAACTGTGAGCGGCGAGCCGCTGTCCGTTTCGTGTCACTGACGCGCCGAAGCTGGTTCGGGGATGCGTCGGGAAGGCCAGGGCAGGGGTGACGACCCGTGAGCCAGGAGACCTGCCTCGACAGATAACGTCCTCCGGCGGGGTGTCCGGTCTGGCCGCTTGCTCAGCGCGACCGGACAAAAGCGCCCGTGCGCGCTCGACCGCGCGCGTCCCGATCAGCCTCGCCAAAACACCGGCAGAGGCTTTTCAAAG ATGTTAATTAATATGCATGGTAC.

The plasmid pRVChyC-6 (60) was cut with MfeI and PacI, and the gBlock segment *metE_*5′UTR was inserted into the plasmid by Gibson assembly. Next, the resulting plasmid was transformed into E. coli DH5α cells and selected on LB-chloramphenicol (Chlor) plates. The resulting Chlor^r^ colonies were then screened by PCR for the insert and verified by Sanger sequencing (Genewiz). The purified plasmid was then transformed into NA1000 cells by electroporation and plated on PYE-Chlor plates. The resulting colonies were screened for mChy fluorescence after induction with vanillate.

**(iii) JS440.** The plasmid pRVMCS-2 (Kan^r^) (60) was transformed into NA1000 cells via electroporation and selected for on PYE-Kan plates.

**(iv) JS441.** The JS441 strain was generated by PCR amplifying the last 500 bp of the β′ RNA polymerase gene into the pYFPC-1 plasmid (60). The insert was PCR amplified from the NA1000 chromosome using Betaprime_forward and Betaprime_reverse primers, and the plasmid pYFPC-1 (60) was PCR amplified by pYFPC_forward and pYFPC_reverse primers. The plasmid was then treated with restriction enzyme DpnI, and the insert was placed into the plasmid by Gibson assembly. Next, the resulting plasmid was transformed into E. coli DH5α cells and selected on LB-spectinomycin (Spec) plates. The resulting Spec^r^ colonies were then screened by colony PCR for the insert and verified by Sanger sequencing (Genewiz). The purified plasmid was then transformed into NA1000 cells by electroporation and plated on PYE-Spec-streptomycin (Strep) plates. The resulting colonies were screened for YFP fluorescence.

PCR primers were as follows: Betaprime_forward, 5′TAATATGCATGGTGTCGACGAGATCCAGGAGG; Betaprime_reverse, 5′-TCTTAAGGTTTCGGCGTCCGAAAGCGC; pYFPC_forward, 5′-GCTTTCGGACGCCGAAACCTTAAGATCTCGAGCTCCG; pYFPC_reverse, 5′-GGATCTCGTCGACACCATGCATATTAATTAAGGCGCC.

### Data availability.

All ribosome profiling raw sequencing reads and normalized read count values of cells grown in M2G are deposited in the NCBI GEO database with accession number GSE126485. PYE ribosome profiling data were collected in reference 36 and were pulled from NCBI GEO database accession number GSE54883. Absolute quantitation values of mRNA translation for both M2G and PYE can be found in Table S1. Ribosome profiling data were compared to the following data sets collected in previous reports. Protein localization data are from reference 26. Tn-seq fitness data are from references 39 and 57. Gene essentiality data are from reference 69. KEGG categories are from reference 70. DNA binding site counts for cell cycle master regulators are from reference 10.
