# Supplementary material for: Absolute Measurements of mRNA Translation in Caulobacter crescentus Reveal Important Fitness Costs of Vitamin B12 Scavenging
Source: mSystems. 2019 May 28;4(4):e00170-19. doi: 10.1128/mSystems.00170-19 (PMC6538847; doi:10.1128/mSystems.00170-19)
Supplement: TABLE S2 [file mSystems.00170-19-st002.docx]

| **M2G** | Ribosome profiling |  | YFP-intensity |  |
| --- | --- | --- | --- | --- |
| Protein | Average molecules of protein translated per cell | σ | Average Proteins per cell | σ |
| B'-YFP | 1.38E+04 | 8.17E+02 | 1.64E+04 | 4.37E+02 |
| CckA-YFP | 7.34E+02 | 6.72E+01 | 6.37E+02 | 1.15E+02 |
| DnaB-YFP | 4.33E+02 | 6.61E+01 | 4.56E+02 | 1.57E+01 |
| HolC-YFP | 4.16E+02 | 7.74E+01 | 3.86E+02 | 9.44E+01 |
| Hu2-YFP | 5.58E+04 | 5.85E+03 | 4.20E+04 | 1.98E+03 |
| L1-YFP | 2.99E+04 | 1.90E+03 | 4.65E+04 | 1.95E+03 |
| MipZ-YFP | 2.41E+03 | 1.13E+02 | 1.03E+03 | 1.91E+02 |
| SMC-YFP | 2.50E+02 | 1.63E+01 | 2.21E+02 | 9.85E+01 |
| TipN-YFP | 9.70E+02 | 9.20E+01 | 6.23E+02 | 4.80E+01 |
| **PYE** | Ribosome profiling*^a^* |  | YFP-intensity |  |
| Protein | Average molecules of protein translated per cell | σ | Average Proteins per cell | σ |
| L1-YFP | 5.36E+04 | ND | 4.65E+04 | 1.39E+03 |
| B'-YFP | 1.64E+04 | ND | 1.64E+04 | 8.22E+01 |
| Ccka-YFP | 8.77E+02 | ND | 6.37E+02 | 1.11E+01 |
| DnaB-YFP | 5.79E+02 | ND | 4.56E+02 | 3.62E+01 |
| HolC-YFP | 3.17E+02 | ND | 3.86E+02 | 1.62E+01 |
| Hu2-YFP | 4.45E+04 | ND | 4.20E+04 | 9.78E+02 |
| Mipz-YFP | 1.03E+03 | ND | 1.03E+03 | 1.33E+01 |
| Smc-YFP | 2.01E+02 | ND | 2.21E+02 | 1.92E+00 |
| TipN-YFP | 3.72E+02 | ND | 6.23E+02 | 1.18E+01 |
