# Supplementary figures and images for: Absolute Measurements of mRNA Translation in Caulobacter crescentus Reveal Important Fitness Costs of Vitamin B12 Scavenging
Source: mSystems. 2019 May 28;4(4):e00170-19. doi: 10.1128/mSystems.00170-19 (PMC6538847; doi:10.1128/mSystems.00170-19)

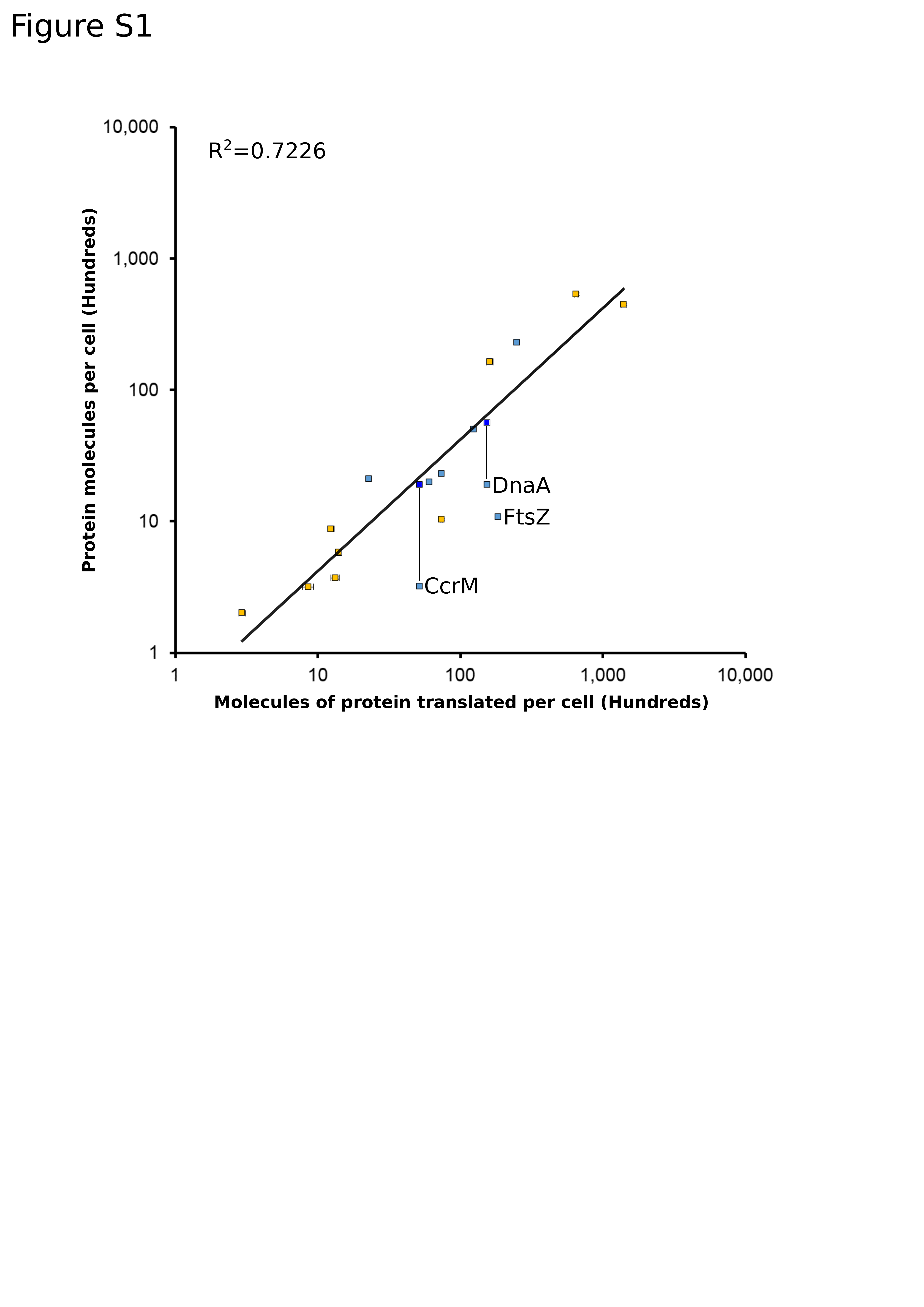

Supplement: FIG S1 [file mSystems.00170-19-sf001.tif]

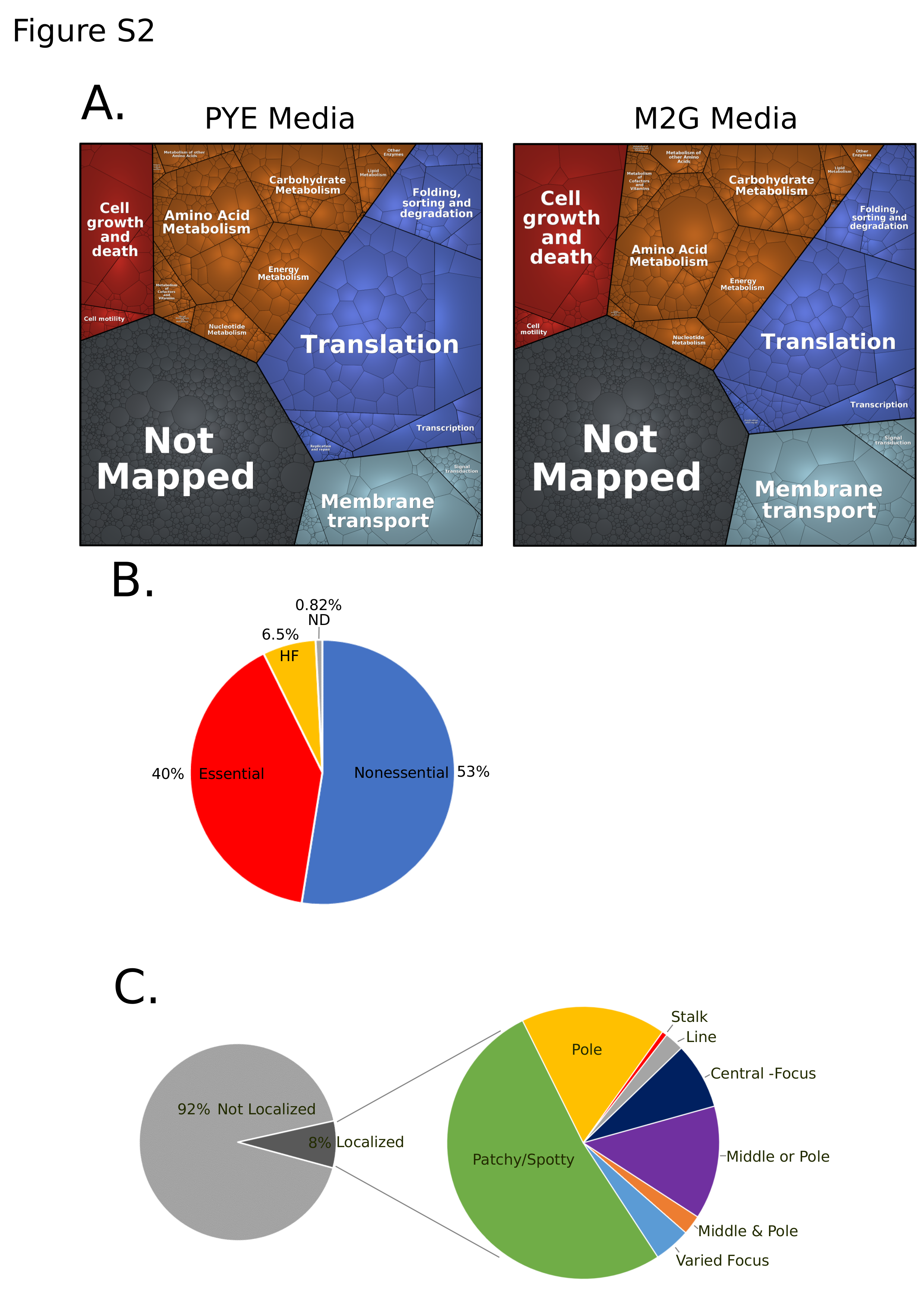

Supplement: FIG S2 [file mSystems.00170-19-sf002.tif]

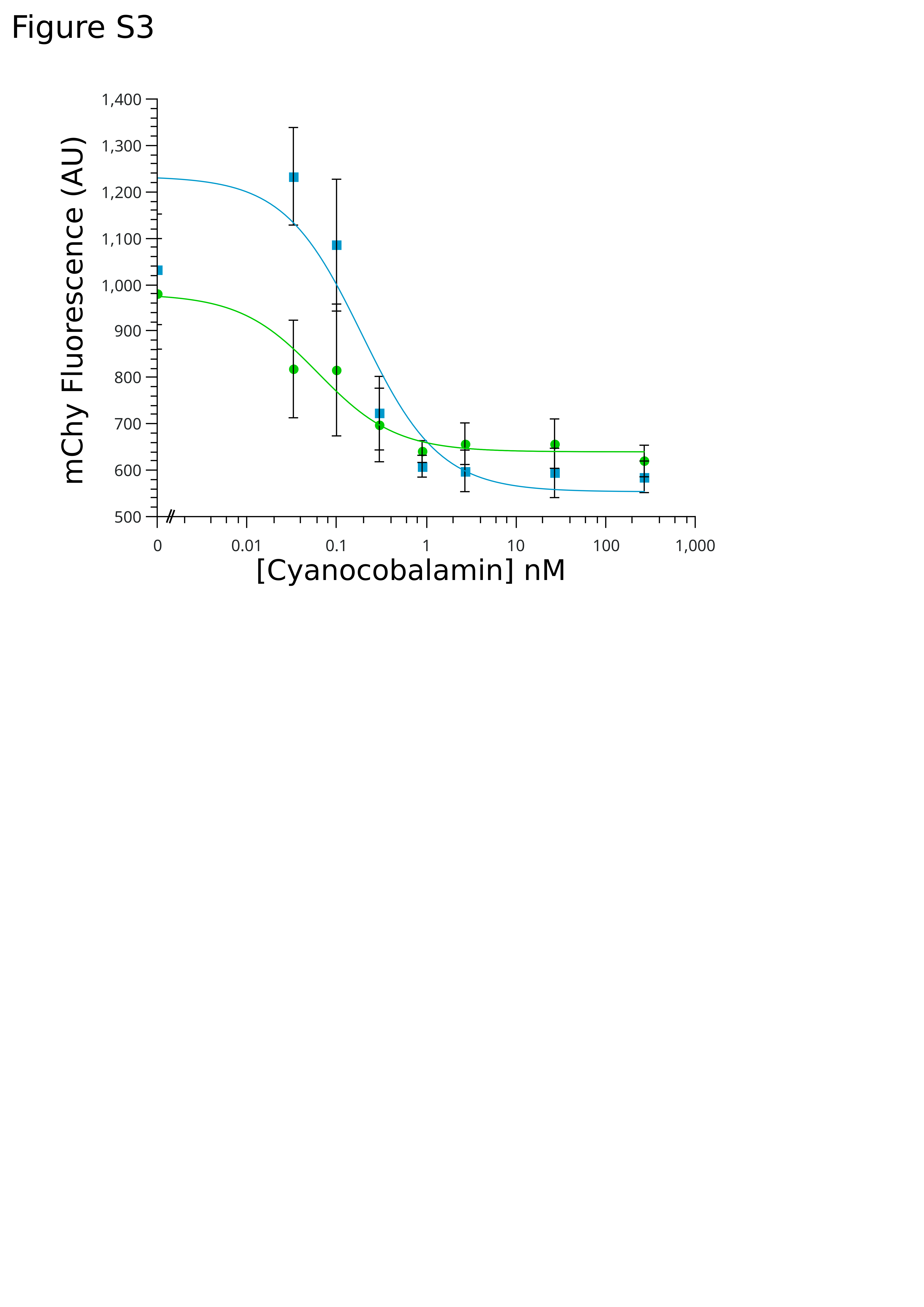

Supplement: FIG S3 [file mSystems.00170-19-sf003.tif]
