# Supplementary material for: Absolute Measurements of mRNA Translation in Caulobacter crescentus Reveal Important Fitness Costs of Vitamin B12 Scavenging
Source: mSystems. 2019 May 28;4(4):e00170-19. doi: 10.1128/mSystems.00170-19 (PMC6538847; doi:10.1128/mSystems.00170-19)
Supplement: TABLE S3 [file mSystems.00170-19-st003.docx]

| Category^a^ | PYE | M2G |  |
| --- | --- | --- | --- |
| Cell growth and death | 6.45% | 11.26% |  |
| Cell motility | 1.19% | 1.09% |  |
| Membrane transport | 9.23% | 11.94% |  |
| Signal transduction | 1.38% | 1.30% |  |
| Folding, sorting and degradation | 5.88% | 5.08% |  |
| Replication and repair | 0.62% | 0.46% |  |
| Transcription | 2.62% | 2.37% |  |
| Translation | 22.82% | 15.88% |  |
| Amino Acid Metabolism | 8.11% | 8.54% |  |
| Carbohydrate Metabolism | 5.51% | 6.09% |  |
| Energy Metabolism | 4.54% | 4.47% |  |
| Glycan Biosynthesis and Metabolism | 0.27% | 0.24% |  |
| Lipid Metabolism | 1.03% | 0.93% |  |
| Metabolism of Cofactors and Vitamins | 1.09% | 1.53% |  |
| Metabolism of other Amino Acids | 0.90% | 0.97% |  |
| Metabolism of Terpenoids and Polyketides | 0.19% | 0.15% |  |
| Nucleotide Metabolism | 1.91% | 1.69% |  |
| Other Enzymes | 0.43% | 0.41% |  |
| Xenobiotics Biodegradation and Metabolism | 0.05% | 0.07% |  |
| Not Mapped | 25.77% | 25.52% |  |
