# Supplementary material for: Absolute Measurements of mRNA Translation in Caulobacter crescentus Reveal Important Fitness Costs of Vitamin B12 Scavenging
Source: mSystems. 2019 May 28;4(4):e00170-19. doi: 10.1128/mSystems.00170-19 (PMC6538847; doi:10.1128/mSystems.00170-19)
Supplement: TABLE S4 [file mSystems.00170-19-st004.docx]

| Gene | Molecules translated per cell | σ | | DNA Sites in cell cycle-regulated promoters | Total DNA binding sites | | References*^a^* |
| --- | --- | --- | --- | --- | --- | --- | --- |
| *dnaA* | 6000 | 459 | 77 | | 84 | (10, 71) | |
| *gcrA* | 18200 | 844 | 94 | | 217 | (10, 24, 72) | |
| *ctrA* | 25400 | 1480 | 183 | | 187 | (10, 73) | |
| *ccrM* | 3280 | 233 | 96 | | 4542 | (10, 74) | |
| *sciP* | 32400 | 1480 | 61 | | 61 | (10, 75) | |
