# Supplementary material for: Absolute Measurements of mRNA Translation in Caulobacter crescentus Reveal Important Fitness Costs of Vitamin B12 Scavenging
Source: mSystems. 2019 May 28;4(4):e00170-19. doi: 10.1128/mSystems.00170-19 (PMC6538847; doi:10.1128/mSystems.00170-19)
Supplement: TABLE S5 [file mSystems.00170-19-st005.docx]

|  | Cyanocobalamin concentration (nM) | | | |
| --- | --- | --- | --- | --- |
|  | 0 | 0.01 | 0.1 | 1 |
| M2G (Minutes) | 135 | 129 | 116 | 111 |
| σ | 1.55 | 1.24 | 2.65 | 2.24 |
|  |  |  |  |  |
| PYE (Minutes) | 101 | 98.1 | 97.8 | 91.2 |
| σ | 1.02 | 1.18 | 1.00 | 0.952 |
