# Supplementary material for: Absolute Measurements of mRNA Translation in Caulobacter crescentus Reveal Important Fitness Costs of Vitamin B12 Scavenging
Source: mSystems. 2019 May 28;4(4):e00170-19. doi: 10.1128/mSystems.00170-19 (PMC6538847; doi:10.1128/mSystems.00170-19)
Supplement: TABLE S6 [file mSystems.00170-19-st006.docx]

| Strain | Genotype | Source |
| --- | --- | --- |
| NA1000 | Synchronizable variant of CB15 | (76) |
| JS417 | NA1000 pRV(*btuB_*5'UTR)CHYC-2 Kan^R^ | This Study |
| JS423 | NA1000 pRV(*metE_*5’UTR)CHYC-6 Chlor^R^ | This Study |
| JS440 | NA1000 pRVMCS-2 Kan^R^ | This Study |
| JS290 | NA1000 *L1::L1-yfp* Gent^R^ | (77) |
| JS441 | NA1000 β′*::* β′*-yfp* Spec^R^ Strep^R^ | This Study |
| NJH429 | NA1000 *cckA::cckA-yfp* RifR | (78) |
| LS3587 | NA1000 dnaB*::dnaB-yfp* Kan^R^ | (79) |
| LS3586 | NA1000 *holC::holC-yfp* Kan^R^ | (79) |
| MS307 | NA1000 *hu2::hu2-yfp* Kan^R^ | (80) |
| MT97 | NA1000 *mipZ::mipZ-yfp* | (62) |
| LS3394 | NA1000 *smc::smc-yfp* Kan^R^ | (81) |
| *tipN-YFP* | NA1000 *tipN::tipN-yfp* | (Gift from Adam Perez) |
