# Supplementary material for: Absolute Measurements of mRNA Translation in Caulobacter crescentus Reveal Important Fitness Costs of Vitamin B12 Scavenging
Source: mSystems. 2019 May 28;4(4):e00170-19. doi: 10.1128/mSystems.00170-19 (PMC6538847; doi:10.1128/mSystems.00170-19)
Supplement: TABLE S7 [file mSystems.00170-19-st007.docx]

| Accession Number | Name*^a^* |
| --- | --- |
| NZ_LSIV00000000 | *Caulobacter* sp. CCH4-E1 |
| NZ_PEGG00000000 | *Caulobacter* sp. B11 |
| NZ_QAIR00000000 | *Caulobacter* sp. HMWF009 |
| NZ_QAII00000000 | *Caulobacter* sp. HMWF025 |
| NZ_AKKF00000000 | *Caulobacter* sp. AP07 |
| NZ_LMHC00000000 | *Caulobacter* sp. Root655 |
| NZ_LMFQ00000000 | *Caulobacter* sp. Root1455 |
| NZ_LMFD00000000 | *Caulobacter* sp. Root487D2Y |
| NC_010338.1 | *Caulobacter* sp. K31 |
| NZ_FORN00000000 | *Caulobacter* sp. UNC279MFTsu5.1 |
| NZ_JONW00000000 | *Caulobacter* sp. UNC358MFTsu5.1 |
| NZ_LMFX00000000 | *Caulobacter* sp. Root1472 |
| NZ_AUEO00000000 | *Caulobacter* sp. URHA0033 |
| NZ_JQJN00000000 | *Caulobacter henricii* strain CF287 |
| NZ_QHJZ00000000 | *Caulobacter* sp. D4A |
| NZ_QHJY00000000 | *Caulobacter* sp. D5 |
| NZ_PJRQ00000000 | *Caulobacter flavus* strain CGMCC1 15093 |
| NZ_QDKQ00000000 | *Caulobacter* sp. 774 |
| NZ_QDKO00000000 | *Caulobacter radicis* strain 695 |
| NZ_PJRS00000000 | *Caulobacter zeae* strain 410 |
| NZ_CP024201 | *Caulobacter mirabilis* strain FWC 38 |
| NZ_CP013002 | *Caulobacter henricii* strain CB4 |
| NZ_JUGJ00000000 | *Caulobacter* sp. OV484 |
| NZ_LMDD00000000 | *Caulobacter* sp. Root342 |
| NZ_LMDF00000000 | *Caulobacter* sp. Root343 |
| NZ_RRYI00000000 | *Caulobacter* sp. 602-1 |
| NZ_PEBF00000000 | *Caulobacter* sp. FWC2 |
| NZ_PEGH00000000 | *Caulobacter* sp. BP25 |
| NZ_LSJA00000000 | *Caulobacter* sp. CCH9-E1 |
| NC_014100 | *Caulobacter segnis* ATCC 21756 |
| NZ_PEGF00000000 | *Caulobacter* sp. X |
| CP001340 | *Caulobacter crescentus* NA1000 |
| NZ_LSJD00000000 | *Caulobacter* sp. CCH5-E12 |
| CP033875 | *Caulobacter* sp. FWC26 |
